# Therapeutic Effect of Tanshinone IIA on Liver Fibrosis and the Possible Mechanism: A Preclinical Meta-Analysis

**DOI:** 10.1155/2019/7514046

**Published:** 2019-12-13

**Authors:** Qingji Ying, Yangyang Teng, Jing Zhang, Zhenzhai Cai, Zhanxiong Xue

**Affiliations:** Department of Gastroenterology, The Second Affiliated Hospital and Yuying Children's Hospital of Wenzhou Medical University, Wenzhou 325000, Zhejiang Province, China

## Abstract

**Background:**

Liver fibrosis is a serious human health problem, and there is a need for specific antifibrosis drugs in the clinic. Tanshinone IIA has recently been reported to have a role in the treatment of liver fibrosis. However, the evidence supporting its antifibrotic effect is not sufficient, and the underlying mechanism is not clear. We thus performed this meta-analysis of animal research to assess the therapeutic effect of tanshinone IIA on liver fibrosis and analyzed the possible associated mechanism to provide a reference for further clinical drug preparation and clinical research.

**Methods:**

We collect related articles from the databases PubMed, Web of Science, Embase, Wanfang, VIP, and CNKI. The quality of the included studies was evaluated according to the SYRCLE risk of bias tool for animal studies. Data were analyzed using RavMan 5.3 and Stata 12.0 software.

**Results:**

A total of 404 articles were retrieved from the databases. After screening, 11 articles were included in the analysis. The included studies' methodological quality was generally low, and an obvious publication bias was found. The results showed that tanshinone IIA significantly improved liver function in experimental animals and reduced the level of liver fibrosis by reducing inflammation and inhibiting immunity, antiapoptotic processes, and HSC activation.

**Conclusion:**

Tanshinone IIA can effectively improve liver fibrosis and liver function in animal models and is worthy of future higher quality animal studies and clinical drug trials.

## 1. Introduction

Liver fibrosis is an important pathological process in the gradual development of various chronic liver diseases, such as viral hepatitis and alcoholic or nonalcoholic fatty liver disease, and it mainly manifests as necrosis in liver cells, excessive deposition of extracellular matrix components, and scar formation by liver fibers. Liver diseases gradually destroy the fibrous mesh scaffolds of the liver, which prevents the liver from reestablishing its normal structure and can cause the liver to lose its normal physiological function, eventually leading to end-stage cirrhosis. Cirrhosis causes approximately 1 million deaths per year [1]. The treatment costs associated with gastrointestinal bleeding, ascites, and other complications are more than three times higher for patients with cirrhosis than for those without cirrhosis [2]. The global burden of liver fibrosis is increasing [3], and drugs to improve liver fibrosis will bring huge benefits.

Liver fibrosis is the early stage of cirrhosis. Pathological biopsy is the gold standard for diagnosing liver fibrosis. The liver fibrosis score is based on liver pathology, and the hydroxyproline (Hyp) content in liver tissue can reflect the degree of liver fibrosis. However, clinically, for noninvasive examinations, serum liver fibrosis markers, such as haluronic acid (HA), laminin (LN), fibrinogen, alanine aminotransferase (ALT), albumin (ALB), and total bilirubin, which are included in this study, are increasingly used to diagnose liver fibrosis [1]. More importantly, their changes in these levels can be used to evaluate fibrosis regression after treatment [4].

Currently, it is believed that effective etiological treatment for liver fibrosis can block the development of fibrosis and even reverse this process [5, 6]. However, over the past two decades, the biology and pathophysiology of hepatic fibrosis have been increasingly understood, and more potential therapeutic targets have been found, providing us with increasingly specific antifibrotic methods. Many antifibrotic drugs have begun clinical trials. However, these antifibrotic agents, such as IL-10, adenosine methionine, ursodeoxycholic acid, silymarin, and colchicine, have not been routinely used in the clinic, and notably, TNF-*α* treatment has been shown to increase infection and mortality rates in patients. Therefore, a product with strong efficacy, high safety, and a low cost is needed for clinical practice [7].

**Figure 1:**
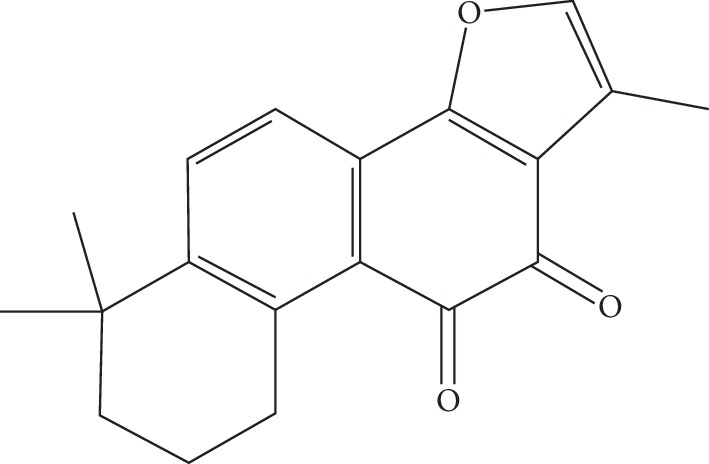
Structure of tanshinone IIA.

The traditional Chinese medicine Salvia miltiorrhiza is made from the root of the lip-shaped plant *Salvia miltiorrhiza*. It is a commonly used in traditional Chinese medicine practices in China. In Chinese medicine theory, Salvia miltiorrhiza belongs to the heart and liver channels and various prescriptions are used to promote blood circulation, calm nerves, regulate menstruation, and relieve pain [8]. Tanshinone IIA is a fat-soluble extract of Salvia miltiorrhiza, and its structure is shown in Figure 1. Recent studies have suggested that tanshinone IIA has anti-inflammatory, antioxidative, antitumor, neuroprotective, and cardioprotective effects in the treatment of atherosclerosis, lung cancer, cervical cancer, Alzheimer's disease, and heart failure [8–12]. Sodium tanshinone IIA sulfonate is a water-soluble sulfonate made from tanshinone IIA, that shows greatly improved water solubility compared with tanshinone IIA, and is more suitable for intravenous infusion. This injectable has been used clinically for the treatment of coronary heart disease for nearly 30 years in China [13]. In recent years, it has been found that tanshinone IIA has a significant effect on organ fibrosis [14–16]. Recently, in vitro studies [17, 18] have suggested that tanshinone IIA can induce activation of hepatic stellate cell (HSC) apoptosis through a variety of pathways, thereby significantly reducing the level of liver fibrosis [19], and may have dose-dependent and time-dependent characteristics [18]. However, the evidence supporting the antifibrotic effect of tanshinone IIA is not sufficient [20], and the underlying mechanism is not clear.

Animal trials are usually performed before clinical trials to evaluate the effectiveness of interventions, which mean that animal studies can be used as the preliminary evidence for the clinical use of drugs. Systematic reviews of animal studies play critical roles in the clarification of physiological and pathological mechanisms in clinical research [21].

Therefore, we conducted this meta-analysis focusing on liver fibrosis animal trials to evaluate tanshinone IIA compared to a placebo in the treatment of liver fibrosis, by assessing levels of change in liver fibrosis markers, particularly with regard to efficacy and the possible mechanism, and to determine whether the results of animal trials of tanshinone IIA can be applied in the clinic.

## 2. Methods

### 2.1. Eligibility Criteria

#### 2.1.1. Studies

Only preclinical animal studies investigating the treatment of liver fibrosis with tanshinone IIA were included in this meta-analysis, regardless of blinding and publication status. Article languages included English and Chinese.

#### 2.1.2. Participants

Animals that successfully model liver fibrosis were included, regardless of the modeling method, age, gender, or species. The diagnosis of liver fibrosis is based on the pathological manifestations of liver tissue.

#### 2.1.3. Intervention and Comparison

Studies which used tanshinone IIA or sodium tanshinone IIA sulfonate as a monotherapy were included. There is no restriction on dosage including frequency, dose, or intensity. Comparator interventions were placebo (inert fluid) or no treatment.

#### 2.1.4. Outcomes

The primary outcome measurements were liver fibrosis index (liver fibrosis score or the level of Hyp, HA, LN, collagen type I, or procollagen type II) and a liver function index (the level of ALT, ALB, or total bilirubin), which can be used to evaluate fibrosis regression after treatment. The secondary outcome measure was a possible mechanism in which tanshinone IIA improves liver fibrosis.

#### 2.1.5. Exclusion Criteria

The publication included previously published results. The full text was not found.

### 2.2. Search Strategies

This meta-analysis follows the PRISMA statement [22]. We searched all the articles on animal experiments evaluating the effects of tanshinone IIA on liver fibrosis in the databases PubMed, Web of Science, Embase, Wanfang, Chinese Scientific Journals Full-Text Database (VIP), and Chinese National Knowledge Infrastructure (CNKI). We searched the databases between inception and 2019.3.20. The article languages included English and Chinese. The search terms included all the keywords such as “Tanshinone” and “Liver Cirrhosis” and free words such as “Tanshinone IIA,” “Tanshinone IIA,” “Cirrhosis, Liver,” “Cirrhoses, Liver,” “Liver Cirrhoses,” “Hepatic Cirrhosis,” “Cirrhoses, Hepatic,” “Cirrhosis, Hepatic,” “Hepatic Cirrhoses,” “Fibrosis, Liver,” “Fibroses, Liver,” “Liver Fibroses,” and “Liver Fibrosis.” The specific search strategies are shown in the supplemental materials.

### 2.3. Study Selection and Data Extraction

According to the eligibility criteria, two authors independently read the titles and abstracts to select potential articles. Then, they independently read the full text of selected articles and made a final decision for selection or not. Discrepancies were discussed and resolved by consensus. We extracted the following data from the full text of the articles: the first author, the year of publication, animal species, animal sex, animal weights, animal numbers, modeling methods, interventions (including method of administration, drug type, and medication timing), anesthesia measures, control group information, and the average and standard deviation of the outcome data. If there were multiple intervention doses evaluated in an experiment, we select only the data for the highest dose. If there were two datasets with different intervention initiation times in the same study, these two datasets were both included. If there were only graphic data such as histograms in the publication, we first contacted the corresponding author of the article. If no response was obtained, the average data and standard deviation analysis were extracted using graphical data extraction software.

### 2.4. Quality Assessment

SYRCLE's risk of bias tool for animal studies [23] was used for evaluations, including (1) whether the allocation sequence was adequately generated and applied, (2) whether the baselines are identical, (3) whether the allocation adequately concealed, (4) whether the animals were randomized during the experiment, (5) whether the researchers were blinded, (6) whether the animals were selected at random for outcome assessment, (7) whether the result evaluators were blinded, (8) whether incomplete data were reported, (9) whether the research report was irrelevant to the selective results report, and (10) whether there were no other biases. “Yes,” “no,” and “uncertainty” represent low bias risk, high bias risk, and uncertain bias risk, respectively.

### 2.5. Data Analysis

We used RavMan 5.3 software for data analysis and Stata 12.0 software for Egger's test and Begg's test. Because of the variability in measurement methods and units among the indicators, direct analysis was expected to produce a high amount of heterogeneity. Therefore, the analysis used the standardized mean difference (SMD) or mean difference (MD) as the effect amount. We used a random-effects model to integrate the effect size. *I*^2^ was used to assess the magnitude of the heterogeneity and to identify potential factors affecting heterogeneity, and we performed a subgroup analysis based on the time when the intervention was given. Therefore, we divided the studies into two groups: the treatment group (the intervention was conducted after modeling) and prevention group (the intervention was conducted before or concurrent with modeling). To assess whether the results were stable, we also conducted a sensitivity analysis.

## 3. Results

### 3.1. Study Inclusion and Characteristics

**Figure 2:**
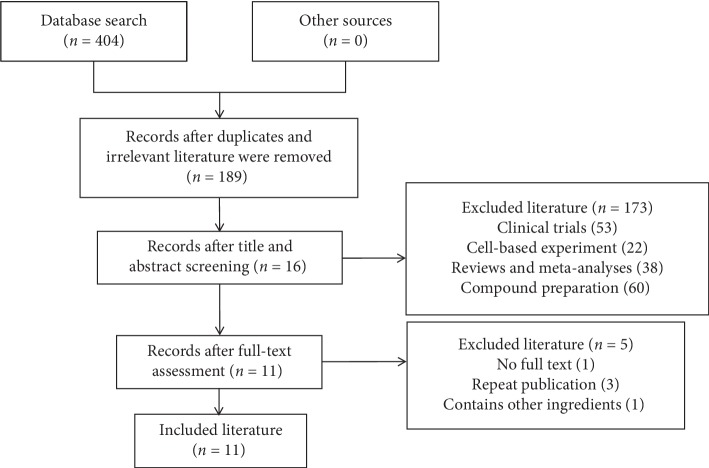
Summary of the process for identifying candidate studies.

We screened a total of 404 articles and were left with 215 articles after removing 189 duplicate or irrelevant documents. Through reading the title and abstract, 173 articles were eliminated. By reading the full text, 5 of the remaining 16 articles were eliminated. Therefore, a total of 11 articles comprising 13 groups of experiments were included [24–34] (Figure 2).

Animal species: eight studies used SD rats [24–26, 29–33]; 1 study used Wistar rats [27]; 1 study used Kunming mice [28]; and 1 study used ICR mice [34]. Three studies used female SD rats [29, 30, 33]; 1 study used a 50 : 50 split of male and female SD rats [25]; and the remaining 7 studies used male animals [24, 26–28, 31, 32, 34]. Anesthesia: three studies used pentobarbital [24, 29, 32]; 2 studies used ether [25, 28]; 1 study used chloral hydrate [24], and 1 study used xylazine and ketamine hydrochloride [33]; none of the other 4 studies clearly named the anesthetics used [26, 30, 31, 34]. Modeling method: six studies used carbon tetrachloride (CCL_4_) modeling [24, 25, 27, 29, 31, 32]; 3 studies used thioacetamide (TAA) modeling [28, 33, 34]; 1 study used pig serum modeling [30]; and 1 study used dimethyl nitrosamine (DMN) modeling [26]. Modeling time: one study ran for 3 weeks [26]; 1 study performed prevention group modeling for 4 weeks, and treatment group modeling for 6 weeks [28]; 3 studies took 6 weeks [27, 31, 32]; 3 studies took for 8 weeks [25, 30, 34]; 2 studies took 12 weeks [24, 29]; and 1 study took 14 weeks [33]. Intervention initiation time: five experiments initiated the intervention before or concurrent with modeling [26–28, 30, 32], while 8 experiments initiated the intervention after modeling [24, 25, 28–31, 33, 34]. Dose: one study treated animals with 2 mg/kg tanshinone IIA [34]; 1 study treated animals with 20 mg/kg tanshinone IIA [33]; 3 studies treated animals with 21.3 mg/kg tanshinone IIA [24, 25, 27]; 1 study treated animals with 100 mg/kg tanshinone IIA [26]; 1 study treated animals with 200 mg/kg tanshinone IIA [29]; 2 studies treated animals with 15 mg/kg sodium tanshinone IIA sulfonate [30, 32]; and 2 studies treated animals with 20 mg/kg sodium tanshinone IIA sulfonate [28, 31]. Administration: six studies utilized intragastric administration [24–27, 29, 32]; 3 studies utilized intraperitoneal injection [28, 30, 31]; 1 study utilized tail vein injection [34]; and 1 study did not explicitly state the mode of administration [33]. The characteristics of the 11 included studies are summarized in detail in Table 1.

### 3.2. Quality Assessment

The overall article methodological quality is summarized in Table 2. All studies only mentioned random allocation; they did not specify the specific randomization methods. The baselines of 3 studies [24, 30, 31] were the same, and none of the remaining studies mentioned baseline evaluations. The randomized allocation of animals was mentioned in 4 studies [24, 25, 31, 33], but not in the rest of the studies. Three studies [29, 30, 32] mentioned the deaths of animals during the modeling process but did not give any explanations on whether the missing data affected the validity of the final results. Two studies [31, 33] did not explicitly mention whether all animals were included in the final analysis, but the remaining studies included all data completely. All the studies fully reported all expected results. None of the studies mentioned whether the researchers were blinded, whether the animals were selected at random for outcome assessment, or whether the evaluators were blinded while analyzing the results, and it was not possible to determine whether there were other biases.

### 3.3. Ameliorative Effects of Tanshinone IIA on Liver Fibrosis

#### 3.3.1. Liver Fibrosis Scores

**Figure 3:**
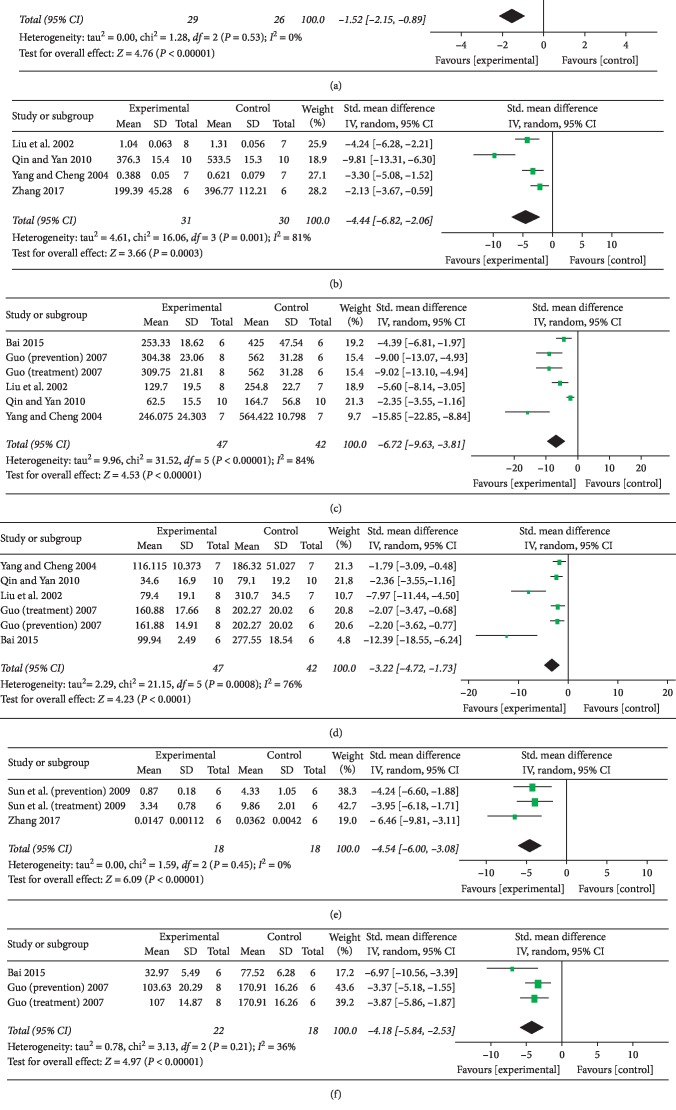
Forest plot: (a) ability of tanshinone IIA to decrease the liver fibrosis score compared with that of control treatment; (b) ability of tanshinone IIA to decrease Hyp content in liver tissue compared with that of control treatment; (c) ability of tanshinone IIA to decrease HA levels compared with that of control treatment; (d) ability of tanshinone IIA to decrease LN levels compared with that of control treatment; (e) ability of tanshinone IIA to decrease collagen type I levels compared with that of control treatment; (f) ability of tanshinone IIA to decrease procollagen type III levels compared with that of control treatment.

A total of 3 studies evaluated the degree of fibrosis in the liver by examining pathological staining of liver sections [25, 32, 34]. The scores ranged from 0 to 4 according to the extent of liver structural damage. The three studies used different criteria, but the criteria were similar. The scoring criteria have been uploaded as a supplement. The tanshinone IIA-treated group showed significantly reduced liver fibrosis scores (*n* = 55, SMD −1.52, 95% CI [−2.15 to −0.89], *P* < 0.01; heterogeneity: *χ*^2^ = 1.28, d*f* = 2 (*P*=0.53); *I*^2^ = 0%) (Figure 3(a)).

#### 3.3.2. Hydroxyproline (Hyp)

Four studies examined the level of Hyp in liver tissue [24, 26, 27, 29], and the level of Hyp in the tanshinone IIA-treated group was significantly lower than that in the model group (*n* = 61, SMD −3.55, 95% CI [−4.52 to −2.58], *P* < 0.01; heterogeneity: *χ*^2^ = 16.06, d*f* = 3 (*P*=0.53); *I*^2^ = 81%) (Figure 3(b)).

#### 3.3.3. Hyaluronic Acid (HA)

Five studies [26, 27, 29–31] showed a significant decrease in the hyaluronic acid level, but the heterogeneity was significant (*n* = 89, SMD −6.72, 95% CI [−9.63 to −3.81], *P* < 0.01; heterogeneity: *χ*^2^ = 31.52, d*f* = 5 (*P* < 0.01); *I*^2^ = 84%) (Figure 3(c)).

#### 3.3.4. Laminin (LN)

In the 5 studies that evaluated hyaluronic acid levels mentioned above [26, 27, 29–31], the laminin level showed a significant decrease in the tanshinone IIA-treated group (*n* = 89, SMD −3.22, 95% CI [−4.72 to −1.73], *P* < 0.01; heterogeneity: *χ*^2^ = 21.15, d*f* = 5 (*P* < 0.01); *I*^2^ = 76%) (Figure 3(d)).

#### 3.3.5. Collagen Type I (Col I) and Procollagen Type III (PCIII)

The levels of serum type I collagen [24, 28] (*n* = 36, SMD −4.54, 95% CI [−6.00 to −3.08], *P* < 0.01; heterogeneity: *χ*^2^ = 1.59, d*f* = 2 (*P*=0.45); *I*^2^ = 0%) (Figure 3(e)) and type III procollagen [30, 31] (*n* = 40, SMD −4.18, 95% CI [−5.84 to −2.53], *P* < 0.01; heterogeneity: *χ*^2^ = 3.13, d*f* = 2 (*P*=0.21); *I*^2^ = 36%) (Figure 3(f)) were significantly lower in the tanshinone IIA-treated group than in the model group.

### 3.4. Ameliorative Effects of Tanshinone IIA on Liver Function

#### 3.4.1. Alanine Aminotransferase (ALT)

**Figure 4:**
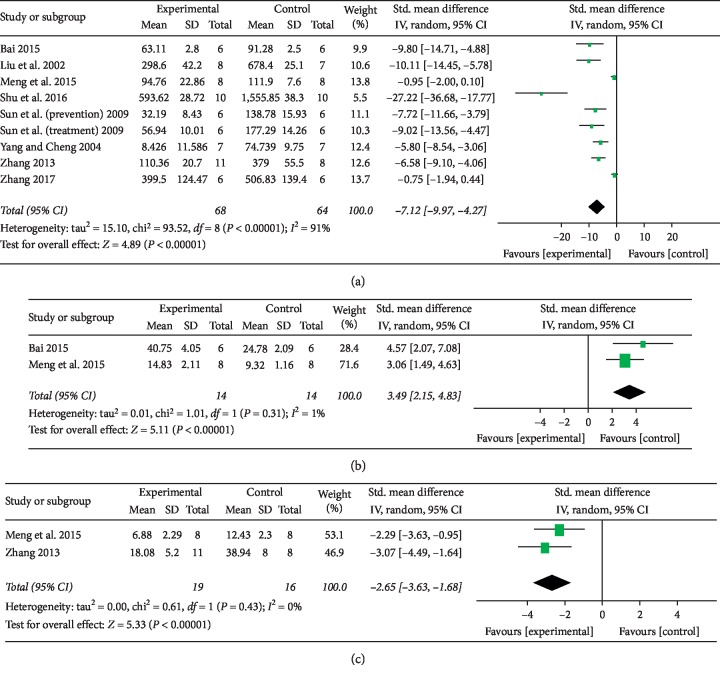
Forest plot: (a) ability of tanshinone IIA to decrease the serum ALT level compared with that of control treatment; (b) ability of tanshinone IIA to increase the serum ALB level compared with that of control treatment; (c) ability of tanshinone IIA to decrease the serum total bilirubin level compared with that of control treatment.

Eight studies [24, 26, 28, 29, 31–34] evaluated serum ALT. The tanshinone IIA-treated group showed a decrease in the ALT level (*n* = 132, SMD −7.12, 95% CI [−9.97 to −4.27], *P* < 0.01; heterogeneity: *χ*^2^ = 93.52, d*f* = 8 (*P* < 0.01); *I*^2^ = 91%) (Figure 4(a)).

#### 3.4.2. Albumin (ALB)

Serum ALB was assessed in 2 studies [31, 34], and the serum albumin level was significantly higher in the tanshinone IIA-treated group than in the model group (*n* = 28, SMD 3.49, 95% CI [2.15 to 4.83], *P* < 0.01; heterogeneity: *χ*^2^ = 1.01, d*f* = 1 (*P*=0.31); *I*^2^ = 1%) (Figure 4(b)).

#### 3.4.3. Total Bilirubin

Two studies [32, 34] reported data for total bilirubin, and there was a significant decrease in the serum total bilirubin level in the tanshinone IIA-treated group (*n* = 35, SMD −2.65, 95% CI [−3.63 to −1.68], *P* < 0.01; heterogeneity: *χ*^2^ = 0.61, d*f* = 1 (*P*=0.43); *I*^2^ = 0%) (Figure 4(c)).

### 3.5. Subgroup Analysis

We conducted a subgroup analysis to assess the source of heterogeneity in the included studies based on the intervention start time. Hyp, HA, LN, and ALT measured were used to divide the data into two groups: the treatment group (the intervention was performed after the model was induced) and the prevention group (the intervention was performed before the model was induced). The levels of all four markers, expect those of Hyp, showed significant decreases in both the treatment and the prevention groups compared with the model control group. Hyp had a *P* value of 0.05 in the prevention group, showing that there was no significant difference between experimental and control groups. Hyp and HA levels showed significant decreases in heterogeneity in the treatment group (Hyp: *I*^2^ from 81% to 62%; HA: *I*^2^ from 84% to 45%), but in the prevention group, the *I*^2^ value remained high. The LN and ALT levels demonstrated that, in the prevention group, heterogeneity was reduced significantly (LN: *I*^2^ from 76% to 0%; ALT: *I*^2^ from 91% to 0%), but in the treatment group, there was no significant change in the *I*^2^ value. The results for the SMD, *P* value, and *I*^2^ are summarized in Table 3.

### 3.6. Sensitivity Analysis

The robustness of the integrated results, which showed heterogeneity >70%, was assessed by sensitivity analysis. Two studies were removed. In one study [27], mice were given tanshinone IIA 3 weeks prior to the start of modeling, which was much earlier than in the other studies. Another study [30] performed modeling with pig serum, while the other studies used CCL_4_. After removing these two studies, only one study was left in the Hyp prevention group, indicating a significant decrease in the Hyp level (*P* < 0.01). The heterogeneity in the ALT level did not change, but the HA level in the prevention group and the LN level in the treatment group showed significant decreases in heterogeneity (HA: *I*^2^ from 91% to 64%; ALT: *I*^2^ from 89% to 34%). The results of sensitivity analysis are summarized in Table 3.

### 3.7. Mechanisms by Which Tanshinone IIA Improves Liver Fibrosis

**Figure 5:**
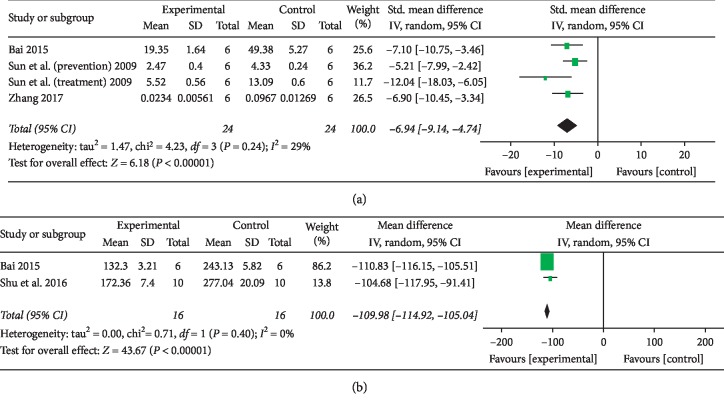
Forest plot: (a) ability of tanshinone IIA to decrease TGF-*β*1 protein expression compared with that of control treatment; (b) ability of tanshinone IIA to decrease TNF-*α* level compared with that of control treatment.

**Figure 6:**
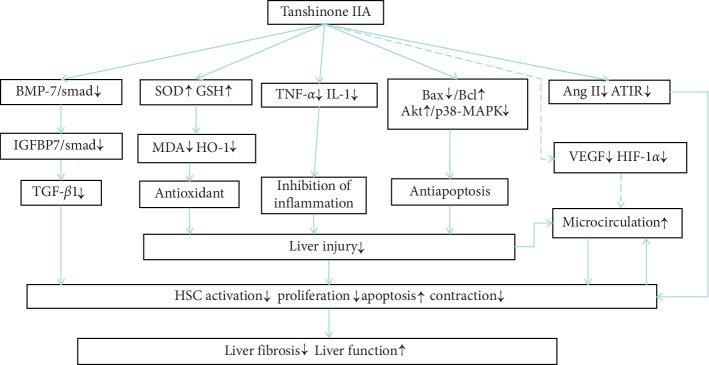
Mechanisms of liver protection mediated by tanshinone IIA in liver fibrosis. Solid lines indicate established effects, whereas dashed lines represent predicted mechanisms.

The results of 3 studies [24, 28, 31] suggested a decrease in TGF-*β*1 protein expression (*n* = 48, SMD −6.94, 95% CI [−9.14 to −4.74], *P* < 0.01; heterogeneity: *χ*^2^ = 4.23, d*f* = 3 (*P*=0.24); *I*^2^ = 29%) (Figure 5(a)). The results of 1 study [25] suggested a significant decrease in TGF-*β*1 mRNA expression (*P* < 0.01). Two studies [31, 33] suggested a decrease in TNF-*α* expression (*n* = 32, SMD −109.98, 95% CI [−114.92 to −105.04], *P* < 0.01; heterogeneity: *χ*^2^ = 0.71, d*f* = 1 (*P*=0.40); *I*^2^ = 0%) (Figure 5(b)). After the sensitivity analysis, 1 study [26] was removed, and only 1 of the remaining studies [33] suggested that SOD and GSH-Px levels were significantly increased (*P* < 0.01) and the MDA level was significantly decreased (*P* < 0.01). One study [24] suggested that the levels of Ang II, AT1R, VEGF, and HIF-1*α* were significantly decreased (*P* < 0.01). One study [32] suggested that the protein expression of Bax was significantly decreased, while the protein expression of Bcl-2 was significantly increased (*P* < 0.01). One study [33] suggested that Akt activation and p38-MAPK were significantly inhibited, while HO-1 expression was significantly decreased (*P* < 0.01). Recent in vitro cytology studies have shown that tanshinone IIA can inhibit TIMP-1 expression and increase MMP-1 expression in HSCs [35], but whether tanshinone IIA can affect liver fibrosis by regulating MMPs and TIMPs in vivo in liver tissue has not been reported in relevant animal experiments. We summarized the mechanism of liver protection mediated by tanshinone IIA in liver fibrosis in Figure 6.

### 3.8. Publication Bias

Due to the small number of studies included, we only used the measurements of ALT to assess publication bias. Through Egger's and Begg's test, we found an obvious publication bias (Egger's *P* value <0.01 and Begg's test *P* value = 0.016). There are many factors that influence the outcome of these tests, not only the nonpublication of negative results but also the heterogeneity among studies, low methodological quality, and having a limited number of small trials [36]. These factors all appeared in this study.

## 4. Discussion

### 4.1. Summary of Evidence

This is the first preclinical meta-analysis of the use of tanshinone IIA in the treatment of animal liver fibrosis. A total of 200 animals were included across 11 studies. According to the evidence, tanshinone IIA can reduce oxidative stress and the liver immune inflammatory response, inhibit liver cell apoptosis, improve the liver microcirculation, inhibit the TGF-*β*1 pathway, reduce the proliferation and activation of HSCs, and ultimately improve liver fibrosis and function. However, the quality of the included articles was generally low, so the results of this meta-analysis should be treated with caution.

### 4.2. Limitations

(1) This meta-analysis only included 11 articles including 9 articles in Chinese and 2 articles in English. The lack of articles in other languages may result in selection bias. None of the included articles mentioned the way in which random allocations were performed. There was no mention of allocation concealment or blinding, so there was a risk of other biases. (2) Many negative results may not be published, and positive results may cause publication bias, resulting in an overestimation of the effect of tanshinone IIA. (3) The results of this meta-analysis show a high degree of heterogeneity. Although some heterogeneity was reduced by sensitivity and subgroup analyses, the heterogeneity within some groups was still high. It is likely that this heterogeneity was related to the insufficient sample size in the included article, which may affect our judgment of the effect of tanshinone IIA. (4) There are articles suggesting that tanshinone IIA does not damage liver cells [17, 37], but no adverse reactions were reported in the included studies. (5) This meta-analysis was not registered so there may be some bias during the research process.

### 4.3. Implications

Liver fibrosis has long plagued clinical practices. Continued progression of any chronic liver disease can lead to liver fibrosis. By reading a number of guidelines for liver disease, it has been found that we can treat liver fibrosis using a variety of traditional Chinese medicine preparations. However, there are currently no large, randomized, multicenter clinical studies being performed to confirm the antifibrotic effects of traditional Chinese medicine preparations. Tanshinone IIA is an extract of Salvia miltiorrhiza, which has antioxidative and anti-inflammatory effects, but its application in liver fibrosis is still lacking. This meta-analysis comprehensively analyzed data from several animal experiments. According to the results, tanshinone IIA reduces liver fiber scores, collagen content in liver tissue, multiple serum fibrosis indexes, and serum liver enzyme levels and restores the serum albumin levels. This analysis also described possible mechanisms related to improving liver fibrosis. The results will provide an important reference for subsequent clinical trials [38].

We utilized the SYRCLE assessment tool for quality assessment. Unlike clinical trials, which are strictly randomized and blinded, most animal experiments do not mention specific methods of distribution [39]. Like the studies we included, all studies do not report the methodology clearly; this deficiency makes us more likely to obtain positive results [40]. The number of samples in animal studies is usually small, and because there is no standard protocol, animal age and sex and experiment duration vary greatly. These shortcomings seriously affect our direct application of animal trial results and meta-analysis of these data [41]. Moreover, for animal experiments, the randomized allocation of animals is relatively important. Lighting and temperature differences during housing have impact on animal behavior, the metabolic rate, and drug toxicity [23]. Therefore, we recommend that subsequent animal trials follow the items in the SYRCLE assessment tool [23] and the ARRIVE Animal Experiment Report [42].

Preclinical animal models are indispensable for identifying novel drug targets for the development of future therapies. The variability among individual models sometimes complicates the comparability of studies and can hamper the translation of results to human diseases [43]. It is important to identify and develop clinically relevant and reliable animal models. The four modeling methods in the studies evaluated here have the disadvantages of high model animal mortality and differences in pathophysiological processes between the model animals and human liver fibrosis. There is currently no ideal animal model for all types of liver fibrosis [44], and different modeling methods must be used for different research purposes. Considering the large number of people infected with hepatitis B virus [3], the incidence of cirrhosis caused by alcoholic or nonalcoholic fatty liver disease has risen sharply [45, 46]; therefore, we recommend that, for HBV-induced liver fibrosis, we can use a primate HBV model or tree scorpion HBV model [47, 48]; for alcohol-induced liver fibrosis, we can use an alcohol-fed mouse model [49]; and for nonalcoholic fatty liver disease-induced liver fibrosis, we can use the iron load supplement diet-fed diabetic mouse model [50]. However, the abovementioned modeling methods are not completely in line with the pathophysiological processes of human liver fibrosis, and there are also ethical and cost-related problems. More ideal models still require subsequent research.

## 5. Conclusion

This meta-analysis suggests that tanshinone IIA may have a therapeutic effect on animal liver fibrosis through its antioxidative, anti-inflammatory, and antiapoptotic properties and its abilities to improve the microcirculation and inhibition of HSC proliferation and activation. Tanshinone IIA is worthy of study in subsequent higher quality animal studies and clinical drug trials.

## Figures and Tables

**Table 1:** Characteristics of the 11 included studies.

First author	Animal species	Number	Modeling methods	Anesthesia	Interventions	Outcome	*P* value
Zhang [24]	Male SD rats	6/6	40% CCl_4_ (2.5 ml/kg) twice a week for 12 weeks subcutaneously	Pentobarbital sodium	Tanshinone IIA (21.3 mg/(kg·d)) for 10 weeks (3–12) intragastrically	(1) Hyp (2) ALT (3) AST (4) Col I (5) ANG II (6) AT1R (7) TGF-*β*1	(1) *P* < 0.05 (2) *P*=0.22 (3) *P*=0.08 (4) *P* < 0.05 (5) *P* < 0.01 (6) *P* < 0.01 (7) *P* < 0.05
Zhang [25]	Male and female SD rats	10/10	10% CCl_4_ (5 ml/kg) for 8 weeks subcutaneously	Ether	Tanshinone IIA (21.3 mg/(kg·d)) for 4 weeks (5–8) intragastrically	(1) Fibrosis score (2) TGF-*β*1 (3) Smad6, 7 (4) BMP7	(1) *P* < 0.01 (2) *P* < 0.01 (3) *P* < 0.01 (4) *P* < 0.01
Yang and Cheng [26]	Male SD rats	7/7	DMN (10 mg/kg) for 3 weeks (3 consecutive days/week)	Not mentioned	Tanshinone IIA (100 mg/kg) for 3 weeks (same time) intraperitoneally	(1) Hyp (2) HA (3) LN (4) ALT (5) MDA (6) SOD (7) GSH-Px	(1) *P* < 0.01 (2) *P* < 0.01 (3) *P* < 0.01 (4) *P* < 0.01 (5) *P* < 0.01 (6) *P*=0.13 (7) *P*=0.13
Qin and Yan [27]	Male Wistar rats	10/10	40% CCl_4_ twice a week for 6 weeks (3–8) (first time 3 ml/kg and then 1 ml/kg) intragastrically	Chloral hydrate	Tanshinone IIA (21.3 mg/(kg·d)) for 8 weeks intragastrically	(1) Hyp (2) HA (3) LN	(1) *P* < 0.01 (2) *P* < 0.01 (3) *P* < 0.01
Sun et al. [28]	Male Kunming mice	Prevention: 6/6 treatment: 6/6	TAA (200 mg/kg) three times a week for 4 weeks (prevention group)/6 weeks (treatment group) intraperitoneally	Ether	Prevention group: sodium tanshinone IIA sulfonate (20 mg/kg) for 4 weeks, intraperitoneally Treatment group: sodium tanshinone IIA sulfonate (20 mg/kg) for 3 weeks (4–6) intraperitoneally	Prevention and treatment group: (1) ALT (2) Col I (3) TGF-*β*1 (4) Smad3 (5) IGFBP7	Prevention and treatment group: (1) *P* < 0.05 (2) *P* < 0.05 (3) *P* < 0.05 (4) *P* < 0.05 (5) *P* < 0.05
Liu et al. [29]	Female SD rats	8/7	CCL_4_ twice a week for l2 weeks (first time pure CCL_4_ (5 ml/kg) and then 20% CCL_4_ (3 ml/kg)), subcutaneously	Pentobarbital	Tanshinone IIA (200 mg/(kg·d)) for 6 weeks (7–12), intragastrically	(1) Hyp (2) HA (3) LN (4) ALT (5) AST (6) MDA (7) NO	(1) *P* < 0.01 (2) *P* < 0.01 (3) *P* < 0.01 (4) *P* < 0.01 (5) *P* < 0.01 (6) *P* < 0.01 (7) *P* < 0.01
Guo [30]	Female SD rats	Prevention: 8/6 treatment: 8/6	Pig serum (0.5 ml) twice a week for 8 weeks intraperitoneally	Not mentioned	Prevention group: sodium tanshinone IIA sulfonate (15 mg/(kg·d)) for 8 weeks intraperitoneally Treatment group: sodium tanshinone IIA sulfonate (15 mg/(kg·d)) for 8 weeks (9–16) intraperitoneally	Prevention and treatment group: (1) HA (2) LN (3) CIV (4) PCIII	(1) *P* < 0.01 (2) *P* < 0.01 (3) *P* < 0.01 (4) *P* < 0.01
Bai [31]	SD rats	6/6	15% CCL_4_ (0.75/kg) three times a week for 6 weeks intraperitoneally	Not mentioned	Sodium tanshinone IIA sulfonate (20 mg/(kg·d)) for 3 days after successful modeling intraperitoneally	(1) ALB (2) HA (3) LN (4) ALT (5) CIV (6) TGF-*β*1 (7) TNF-*α* (8) PCIII	(1) *P* < 0.01 (2) *P* < 0.01 (3) *P* < 0.01 (4) *P* < 0.01 (5) *P* < 0.01 (6) *P* < 0.01 (7) *P* < 0.01 (8) *P* < 0.01
Zhang [32]	Male SD rats	11/8	50% CCL_4_ (1 ml/kg) twice a week for 6 weeks intragastrically	Pentobarbital sodium	Sodium tanshinone IIA sulfonate injection (15 ml/(kg·d)) for 6 weeks intraperitoneally	(1) Fibrosis score (2) Total bilirubin (3) ALT (4) AST (5) Bax (6) Bcl-2	(1) *P* < 0.01 (2) *P* < 0.01 (3) *P* < 0.01 (4) *P* < 0.01 (5) *P* < 0.01 (6) *P* < 0.01
Shu et al. [33]	Female SD rats	10/10	Drink TAA solution (0.03%) for 14 weeks after ligation of the left superior renal vein	Xylazine, ketamine hydrochloride	Tanshinone IIA (20 mg/(kg·d)) for 3 days after modeling	(1) ALT (2) AST (3) MDA (4) SOD (5) GSH-Px (6) TNF-*α* (7) HO-1 (8) NF-κb (9) IL-1*β* (10) IL-6 (11) Akt (12) p38-MAPK	(1) *P* < 0.01 (2) *P* < 0.01 (3) *P* < 0.01 (4) *P* < 0.01 (5) *P* < 0.01 (6) *P* < 0.01 (7) *P* < 0.01 (8) *P* < 0.01 (9) *P* < 0.01 (10) *P* < 0.01 (11) *P* < 0.01 (12) *P* < 0.01
Meng et al. [34]	Male ICR mice	8/8	TAA (200ug/kg) three times a week for 8 weeks intraperitoneally	Not mentioned	Tanshinone IIA (2 mg/kg) (next day after TAA) for 3 weeks (6–8), injected into the tail vein	(1) Fibrosis score (2) ALB (3) Total bilirubin (4) ALT (5) AST	(1) *P* < 0.01 (2) *P* < 0.01 (3) *P* < 0.01 (4) *P* < 0.01 (5) *P* < 0.01

Hyp, hydroxyproline; ALT, alanine aminotransferase; AST, aspartate aminotransferase; HA, haluronic acid; Col I, collagen type I; Ang II, angiotensin II; AT1R, angiotensin type 1 receptor; TGF-*β*1, transforming growth factor-*β*1; BMP7, bone morphogenetic protein 7; LN, laminin; MDA, malondialdehyde; SOD, superoxide dismutase; GSH-Px, glutathione peroxidase; IGFBP7, insulin-like growth factor-binding protein 7; NO, nitric oxide; CIV, collage type IV; TNF-*α*, tumor necrosis factor-*α*; Bax, bcl-2-associated *x*; Bcl-2, b-cell lymphoma-2; HO-1, heme oxygenase-1; NF-*κ*b, nuclear factor kappa-B; IL-1*β*, interleukin-1*β*; IL-6, interleukin-6; Akt, protein kinase b; ALB, albumin; PCIII, procollagen type III. Prevention group: intervention was conducted before or the same time as modeling; treatment group: intervention was conducted after modeling.

**Table 2:** Risk of bias of the included studies.

	1	2	3	4	5	6	7	8	9	10
Zhang [24]	U	Y	U	Y	U	U	U	Y	Y	U
Zhang [25]	U	U	U	Y	U	U	U	Y	Y	U
Yang and Cheng [26]	U	U	U	U	U	U	U	Y	Y	U
Qin and Yan [27]	U	U	U	U	U	U	U	Y	Y	U
Sun et al. [28]	U	U	U	U	U	U	U	Y	Y	U
Liu et al. [29]	U	U	U	U	U	U	U	N	Y	U
Guo [30]	U	Y	U	U	U	U	U	N	Y	U
Bai [31]	U	Y	U	Y	U	U	U	U	Y	U
Zhang [32]	U	U	U	U	U	U	U	N	Y	U
Shu et al. [33]	U	U	U	Y	U	U	U	U	Y	U
Meng et al. [34]	U	U	U	U	U	U	U	Y	Y	U

Y, yes; N, no; U, unclear; (1) whether the allocation sequence adequately generated and applied; (2) whether the baselines are identical; (3) whether the allocation adequately concealed; (4) whether the animals were randomly placed during the experiment; (5) whether researchers were blinded; (6) whether the animals were selected at random for outcome assessment; (7) whether results evaluators are blinded; (8) whether incomplete data are reported; (9) whether the research report is irrelevant to the selective results report; (10) whether there is no other bias.

**Table 3:** Subgroup and sensitivity analysis of indicators.

	Hyp			HA			LN			ALT		
	SMD (95% CI)	*P*	*I* ^2^ (%)	SMD (95% CI)	*P*	*I* ^2^ (%)	SMD (95% CI)	*P*	*I* ^2^ (%)	SMD (95% CI)	*P*	*I* ^2^ (%)
*Subgroups*												
Treatment group	−3.08 (−5.14, −1.02)	<0.01	62	−5.86 (−8.12, −3.59)	<0.01	45	−6.92 (−12.76, −1.08)	0.02	89	−7.45 (−11.10, −3.80)	<0.01	93
Prevention group	−6.37 (−12.74, 0.0)	0.05	91	−8.41 (−15.65, −1.18)	0.02	91	−2.21 (−2.87, −1.37)	<0.01	0	−6.49 (−8.17, −4.82)	<0.01	0
Overall	−4.44 (−6.82, −2.06)	<0.01	81	−6.72 (−9.63, −3.81)	<0.01	81	−3.22 (−4.72, −1.73)	<0.01	76	−7.12 (−9.97, −4.27)	<0.01	91

*Sensitivity analysis*												
Treatment group	−3.08 (−5.14, −1.02)	<0.01	62	−5.86 (−8.12, −3.59)	<0.01	45	−9.42 (−13.49, −5.35)	<0.01	34	−7.45 (−11.10, −3.80)	<0.01	93
Prevention group	−3.30 (−5.08, −1.52)	<0.01	—	−11.81 (−18.41, −5.21)	<0.01	64	−2.10 (−2.98, −1.21)	<0.01	0	−6.49 (−8.17, −4.82)	<0.01	0

## Supplementary Materials

Supplementary Materials PRISMA 2009 Checklist.
