# Supplementary material for: Therapeutic Effect of Tanshinone IIA on Liver Fibrosis and the Possible Mechanism: A Preclinical Meta-Analysis
Source: Evid Based Complement Alternat Med. 2019 Dec 13;2019:7514046. doi: 10.1155/2019/7514046 (PMC6930756; doi:10.1155/2019/7514046)
Supplement: Supplementary Materials — PRISMA 2009 Checklist. [file 7514046.f1.pdf]

PRISMA 2009 Checklist

Section/topic	#	Checklist item	Reported on page #
**TITLE**			
Title	1	Identify the report as a systematic review, meta-analysis, or both.	1
**ABSTRACT**			
Structured summary	2	Provide a structured summary including, as applicable: background; objectives; data sources; study eligibility criteria, participants, and interventions; study appraisal and synthesis methods; results; limitations; conclusions and implications of key findings; systematic review registration number.	1-2
**INTRODUCTION**			
Rationale	3	Describe the rationale for the review in the context of what is already known.	2-4
Objectives	4	Provide an explicit statement of questions being addressed with reference to participants, interventions, comparisons, outcomes, and study design (PICOS).	2-5
**METHODS**			
Protocol and registration	5	Indicate if a review protocol exists, if and where it can be accessed (e.g., Web address), and, if available, provide registration information including registration number.	18
Eligibility criteria	6	Specify study characteristics (e.g., PICOS, length of follow-up) and report characteristics (e.g., years considered, language, publication status) used as criteria for eligibility, giving rationale.	5-6
Information sources	7	Describe all information sources (e.g., databases with dates of coverage, contact with study authors to identify additional studies) in the search and date last searched.	6-7
Search	8	Present full electronic search strategy for at least one database, including any limits used, such that it could be repeated.	6-7
Study selection	9	State the process for selecting studies (i.e., screening, eligibility, included in systematic review, and, if applicable, included in the meta-analysis).	7
Data collection process	10	Describe method of data extraction from reports (e.g., piloted forms, independently, in duplicate) and any processes for obtaining and confirming data from investigators.	7
Data items	11	List and define all variables for which data were sought (e.g., PICOS, funding sources) and any assumptions and simplifications made.	7
Risk of bias in individual studies	12	Describe methods used for assessing risk of bias of individual studies (including specification of whether this was done at the study or outcome level), and how this information is to be used in any data synthesis.	7
Summary measures	13	State the principal summary measures (e.g., risk ratio, difference in means).	8
Synthesis of results	14	Describe the methods of handling data and combining results of studies, if done, including measures of consistency (e.g., I^2) for each meta-analysis.	8

PRISMA 2009 Checklist

Section/topic	#	Checklist item	Reported on page #
Risk of bias across studies	15	Specify any assessment of risk of bias that may affect the cumulative evidence (e.g., publication bias, selective reporting within studies).	8
Additional analyses	16	Describe methods of additional analyses (e.g., sensitivity or subgroup analyses, meta-regression), if done, indicating which were pre-specified.	8
**RESULTS**			
Study selection	17	Give numbers of studies screened, assessed for eligibility, and included in the review, with reasons for exclusions at each stage, ideally with a flow diagram.	8-9
Study characteristics	18	For each study, present characteristics for which data were extracted (e.g., study size, PICOS, follow-up period) and provide the citations.	9-10
Risk of bias within studies	19	Present data on risk of bias of each study and, if available, any outcome level assessment (see item 12).	10
Results of individual studies	20	For all outcomes considered (benefits or harms), present, for each study: (a) simple summary data for each intervention group (b) effect estimates and confidence intervals, ideally with a forest plot.	11-14
Synthesis of results	21	Present results of each meta-analysis done, including confidence intervals and measures of consistency.	11-14
Risk of bias across studies	22	Present results of any assessment of risk of bias across studies (see Item 15).	17
Additional analysis	23	Give results of additional analyses, if done (e.g., sensitivity or subgroup analyses, meta-regression [see Item 16]).	14-15
**DISCUSSION**			
Summary of evidence	24	Summarize the main findings including the strength of evidence for each main outcome; consider their relevance to key groups (e.g., healthcare providers, users, and policy makers).	17-18
Limitations	25	Discuss limitations at study and outcome level (e.g., risk of bias), and at review-level (e.g., incomplete retrieval of identified research, reporting bias).	18
Conclusions	26	Provide a general interpretation of the results in the context of other evidence, and implications for future research.	19-20
**FUNDING**			
Funding	27	Describe sources of funding for the systematic review and other support (e.g., supply of data); role of funders for the systematic review.	21

From: Moher D, Liberati A, Tetzlaff J, Altman DG, The PRISMA Group (2009). Preferred Reporting Items for Systematic Reviews and Meta-Analyses: The PRISMA Statement. PLoS Med 6(6): e1000097. doi:10.1371/journal.pmed1000097

For more information, visit: www.prisma-statement.org.

Database search strategy

The search strategy in Pubmed: ("tanshinone"[Supplementary Concept] OR (("tanshinone"[Supplementary Concept] OR "tanshinone"[All Fields] OR "tanshinone ii a"[All Fields]) OR ("tanshinone"[Supplementary Concept] OR "tanshinone"[All Fields] OR "tanshinone iia"[All Fields]))) AND ("liver cirrhosis"[MeSH Terms] OR (((((((((("liver cirrhosis"[MeSH Terms] OR ("liver"[All Fields] AND "cirrhosis"[All Fields]) OR "liver cirrhosis"[All Fields] OR ("cirrhosis"[All Fields] AND "liver"[All Fields]) OR "cirrhosis, liver"[All Fields]) OR ("liver cirrhosis"[MeSH Terms] OR ("liver"[All Fields] AND "cirrhosis"[All Fields]) OR "liver cirrhosis"[All Fields] OR ("cirrhoses"[All Fields] AND "liver"[All Fields]))) OR ("liver cirrhosis"[MeSH Terms] OR ("liver"[All Fields] AND "cirrhosis"[All Fields]) OR "liver cirrhosis"[All Fields] OR ("liver"[All Fields] AND "cirrhoses"[All Fields]) OR "liver cirrhoses"[All Fields])) OR ("liver cirrhosis"[MeSH Terms] OR ("liver"[All Fields] AND "cirrhosis"[All Fields]) OR "liver cirrhosis"[All Fields] OR ("hepatic"[All Fields] AND "cirrhosis"[All Fields]) OR "hepatic cirrhosis"[All Fields])) OR ("liver cirrhosis"[MeSH Terms] OR ("liver"[All Fields] AND "cirrhosis"[All Fields]) OR "liver cirrhosis"[All Fields] OR ("cirrhoses"[All Fields] AND "hepatic"[All Fields]))) OR ("liver cirrhosis"[MeSH Terms] OR ("liver"[All Fields] AND "cirrhosis"[All Fields]) OR "liver cirrhosis"[All Fields] OR ("cirrhosis"[All Fields] AND "hepatic"[All Fields]) OR "cirrhosis, hepatic"[All Fields])) OR ("liver cirrhosis"[MeSH Terms] OR ("liver"[All Fields] AND "cirrhosis"[All Fields]) OR "liver cirrhosis"[All Fields] OR ("hepatic"[All Fields] AND "cirrhoses"[All Fields]) OR "hepatic cirrhoses"[All Fields])) OR ("liver cirrhosis"[MeSH Terms] OR ("liver"[All Fields] AND "cirrhosis"[All Fields]) OR "liver cirrhosis"[All Fields] OR ("fibrosis"[All Fields] AND "liver"[All Fields]) OR "fibrosis, liver"[All Fields])) OR ("liver cirrhosis"[MeSH Terms] OR ("liver"[All Fields] AND "cirrhosis"[All Fields]) OR "liver cirrhosis"[All Fields] OR ("fibroses"[All Fields] AND "liver"[All Fields]))) OR ("liver cirrhosis"[MeSH Terms] OR ("liver"[All Fields] AND "cirrhosis"[All Fields]) OR "liver cirrhosis"[All Fields] OR ("liver"[All Fields] AND "fibroses"[All Fields]) OR "liver fibroses"[All Fields])) OR ("liver cirrhosis"[MeSH Terms] OR ("liver"[All Fields] AND "cirrhosis"[All Fields]) OR "liver cirrhosis"[All Fields] OR ("liver"[All Fields] AND "fibrosis"[All Fields]) OR "liver fibrosis"[All Fields])))

The search strategy in WOS: TS=(tanshinone or tanshinone II A or tanshinone IIA) and TS=(Liver Cirrhosis or Cirrhosis, Liver or Cirrhoses, Liver or Liver Cirrhoses or Hepatic Cirrhosis or Cirrhoses, Hepatic or Cirrhosis, Hepatic or Hepatic Cirrhoses or Fibrosis, Liver or Fibroses, Liver or Liver Fibroses or Liver Fibrosis)

The search strategy in Embase: (‘tanshinone’/exp or ‘tanshinone II A’ or ‘tanshinone IIA’) and (‘Liver Cirrhosis’/exp or ‘Cirrhosis, Liver’ or ‘Cirrhoses, Liver’ or ‘Liver Cirrhoses’ or ‘Hepatic Cirrhosis’ or ‘Cirrhoses, Hepatic’ or ‘Cirrhosis, Hepatic’ or ‘Hepatic Cirrhoses’ or ‘Fibrosis, Liver’ or ‘Fibroses, Liver’ or ‘Liver Fibroses’ or ‘Liver Fibrosis’)

The search strategy in wanfang: (丹参酮 or 丹参酮 IIA) and (肝纤维化 or 肝硬化)

The search strategy in VIP: K=(丹参酮 OR 丹参酮 IIA) AND K=(肝纤维化 OR 肝硬化)

The search strategy in CNKI: SU=(‘丹参酮’+’丹参酮 IIA’) AND SU=(‘肝纤维化’+’肝硬化’)

Fibrosis scoring criteria

Zhang CH 2015: 0, normal; 1, fibrosis present (collagen fiber present that extends from portal triad or central vein to peripheral region); 2, mild fibrosis (mild collagen fiber present with extension without compartment formation); 3, moderate fibrosis (moderate collagen fiber present with some pseudo lobe formation); 4, severe fibrosis (severe collagen fiber present with thickening of the partial compartments and frequent pseudo lobe formation).

Meng Z 2015: 0, no scarring; 1, minimal scarring; 2, scarring had occurred and extended outside the areas in the liver that contained blood vessels; 3, bridging fibrosis was spreading and connecting to other areas than contained fibrosis; 4, advanced scarring of the liver or cirrhosis.

Zhang M 2013: 0, normal; 1, fibrosis Including and around the portal area, limited sinus fibrosis or fibrous scarring in the lobes; 2, fiber spacing formation (bridge fiberization); 3, a large number of interfiber spaces separate and destroy the hepatic lobules, resulting in a disordered lobular structure; 4, liver cirrhosis, extensive destruction of liver parenchyma, diffuse fibrosis, separated hepatocyte masses with varying degrees of regeneration and pseudolobule formation.
